# Supplementary figures and images for: Immunogenicity and inflammatory properties of respiratory syncytial virus attachment G protein in cotton rats
Source: PLoS One. 2021 Feb 18;16(2):e0246770. doi: 10.1371/journal.pone.0246770 (PMC7891763; doi:10.1371/journal.pone.0246770)

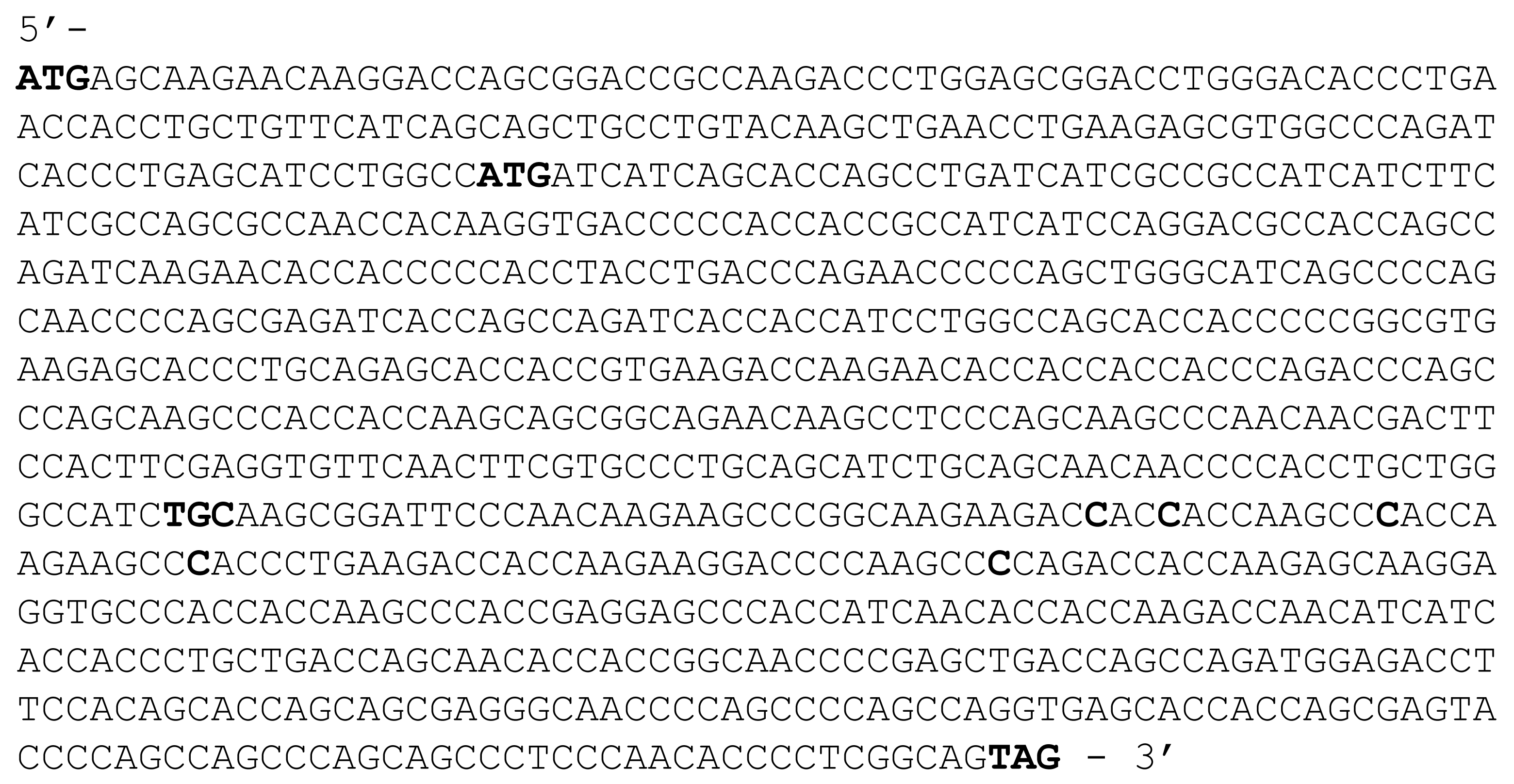

Supplement: S1 Fig — Single bolded letters (C) represent nucleotides that were mutated for codon optimization. A series of three bolded letters represent key sites within the G protein gene that were mutated to produce various G protein constructs to be inserted into the AAV vector. (TIF) [file pone.0246770.s001.tif]

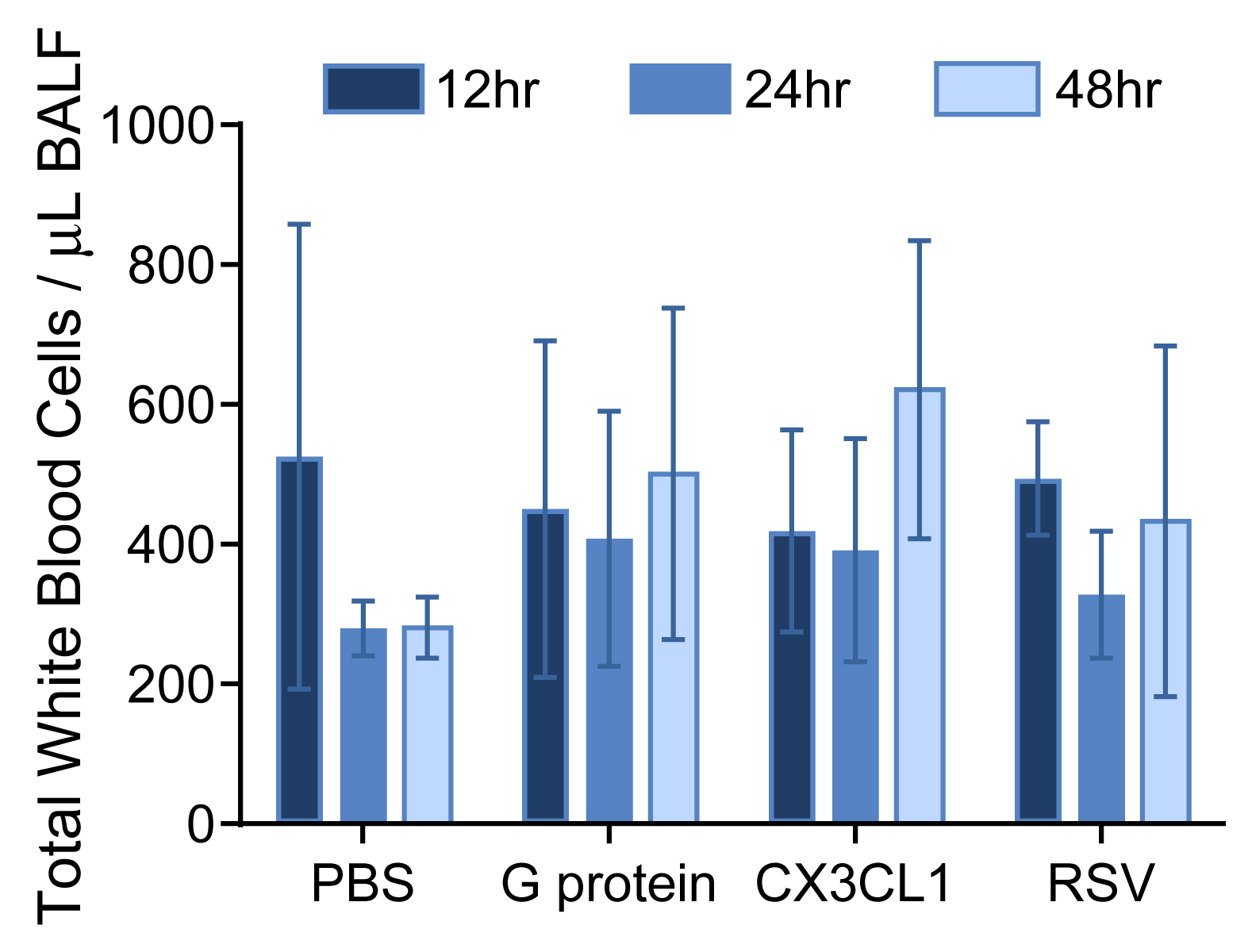

Supplement: S2 Fig — The total number of white blood cells per μL of bronchoalveolar lavage fluid (BALF) were compared at 12 hours, 24 hours, and 48 hours post-inoculation with 100μL of PBS, 50μg/mL purified G protein, 50μg/mL mouse CX3CL1, or 105 TCID50 RSV. Bars and brackets represent the mean and standard deviations. There was no significant difference between groups; Two-way ANOVA; p> 0.05. PBS (n = 3–4), G protein (n = 4), CX3CL1 (n = 4), RSV (n = 2–4). (TIF) [file pone.0246770.s002.tif]

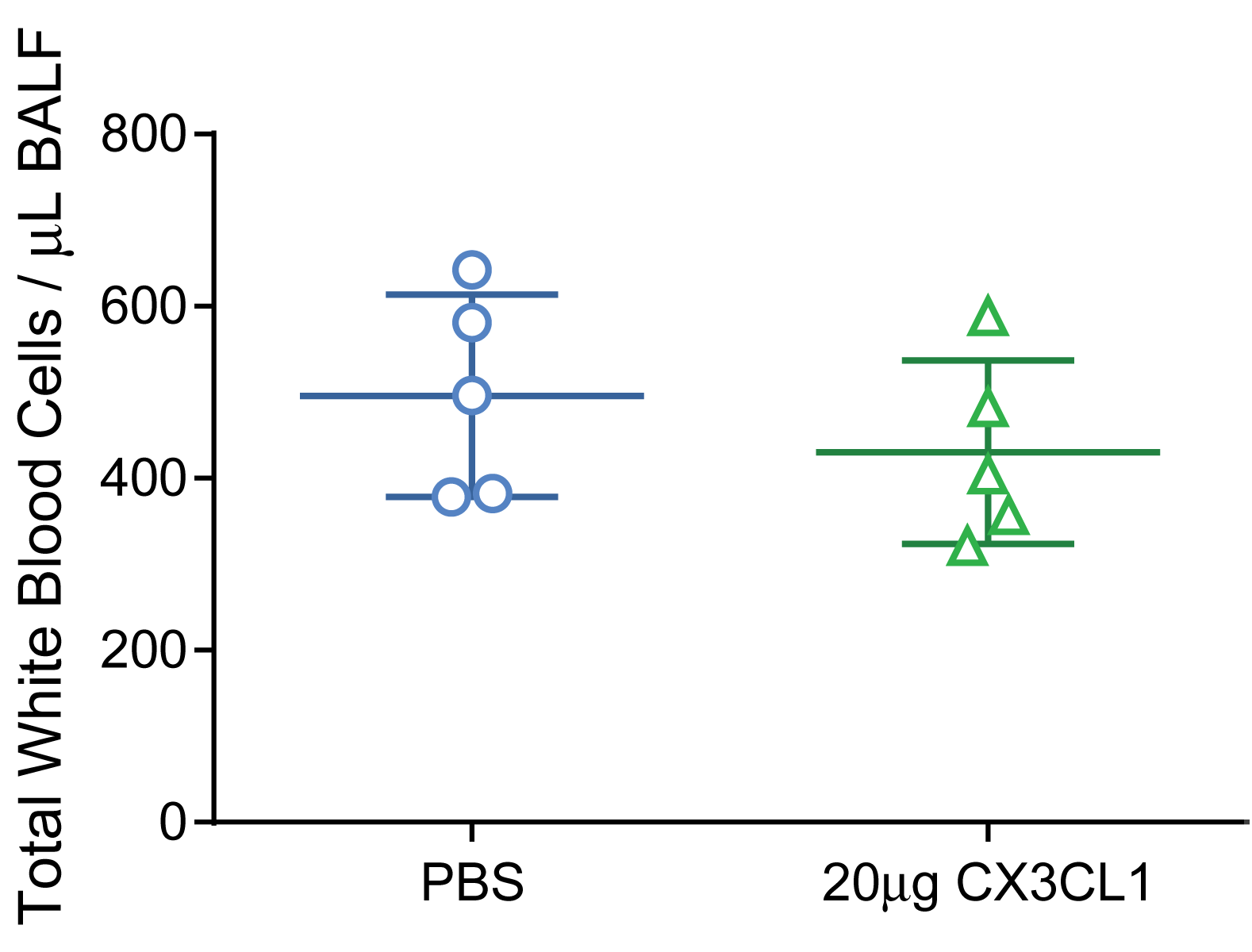

Supplement: S3 Fig — The total number of white blood cells per μL of bronchoalveolar lavage fluid (BALF) were compared 48 hours post-inoculation with 100μL of PBS and 200μg/mL mouse CX3CL1 (n = 5). Bars and brackets represent the mean and standard deviations. There was no significant difference between groups; Student unpaired t test; p> 0.05. (TIF) [file pone.0246770.s003.tif]

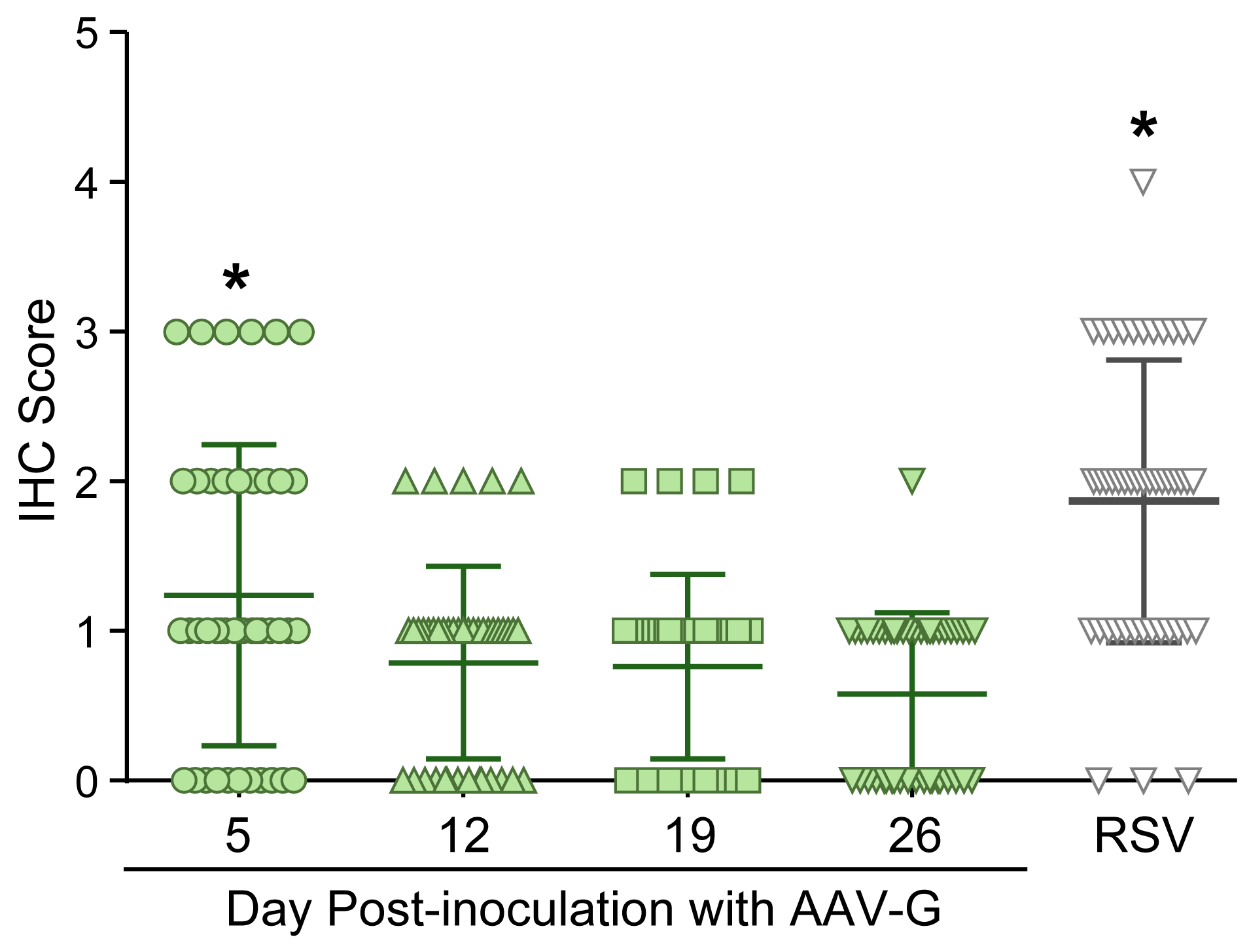

Supplement: S4 Fig — The semi-quantitative scoring of G protein expression via immunohistochemistry (IHC) with an anti-RSV antiserum in lung sections of cotton rats were compared. The mean and standard deviations are represented (n = 42-45/group). The asterisk indicates a significantly higher IHC score compared to all other groups (One-way ANOVA, p<0.05). (TIF) [file pone.0246770.s004.tif]
