# Supplementary material for: Immunogenicity and inflammatory properties of respiratory syncytial virus attachment G protein in cotton rats
Source: PLoS One. 2021 Feb 18;16(2):e0246770. doi: 10.1371/journal.pone.0246770 (PMC7891763; doi:10.1371/journal.pone.0246770)
Supplement: S1 Table — (DOCX) [file pone.0246770.s005.docx]

**S1 Table. Semi-Quantitative Immunohistochemistry Scoring System**

| **Characteristic** | **Description** | **Grade** |
| --- | --- | --- |
| **Percentage of bronchiolar epithelial positive** | 0% bronchiolar epithelium positively stained | 0 |
|  | 0-5% cells positively stained | 1 |
|  | 5-10% positively stained | 2 |
|  | 10-25% positively stained | 3 |
|  | >25% positively stained | 4 |
| **Percentage of alveolar epithellium positive** | 0% alveolar epithelium positively stained | 0 |
|  | 0-5% positively stained | 1 |
|  | 5-10% positively stained | 2 |
|  | 10-25% positively stained | 3 |
|  | >25% positively stained | 4 |
| **Percentage of strong positive staining out of all staining** | 0% positively stained | 0 |
|  | 0-25% positively stained | 1 |
|  | 25-50% positively stained | 2 |
|  | 50-75% positively stained | 3 |
|  | >75% positively stained | 4 |
