# Supplementary material for: Immunogenicity and inflammatory properties of respiratory syncytial virus attachment G protein in cotton rats
Source: PLoS One. 2021 Feb 18;16(2):e0246770. doi: 10.1371/journal.pone.0246770 (PMC7891763; doi:10.1371/journal.pone.0246770)
Supplement: S2 Table — (DOCX) [file pone.0246770.s006.docx]

**S2 Table. Semi-Quantitative Histologic Inflammatory Scoring System**

| **Characteristic** | **Description** | **Grade** |
| --- | --- | --- |
| **Quantity of peribronchiolar infiltrates** | 0% of bronchioles with peribronchiolar infiltrates | 0 |
|  | 0-25% of bronchioles with peribronchiolar infiltrates | 1 |
|  | 25-50% of bronchioles with peribronchiolar infiltrates | 2 |
|  | 50-75% of bronchioles with peribronchiolar infiltrates | 3 |
|  | >75% of bronchioles with peribronchiolar infiltrates | 4 |
| **Severity of peribronchiolar infiltrates** | No cells | 0 |
|  | interrupted cuff | 1 |
|  | Complete cuff, <5 cell layers thick | 2 |
|  | Complete cuff, >5 cell layers thick | 3 |
| **Quantity of bronchiolar infiltrates** | 0% of bronchioles with bronchiolar epithelium infiltrates | 0 |
|  | 0-25% of bronchioles with bronchiolar epithelium infiltrates | 1 |
|  | 25-50% of bronchioles with bronchiolar epithelium infiltrates | 2 |
|  | 50-75% of bronchioles with bronchiolar epithelium infiltrates | 3 |
|  | >75% of bronchioles with bronchiolar epithelium infiltrates | 4 |
| **Severity of bronchiolar infiltrates** | No infiltrating cells within the bronchiolar epithelium | 0 |
|  | 0-10% of the bronchiolar epithelium has inflammatory infiltrates | 1 |
|  | 10-33% of the bronchiolar epithelium has inflammatory infiltrates | 2 |
|  | 33-66% of the bronchiolar epithelium has inflammatory infiltrates | 3 |
|  | >66% of the bronchiolar epithelium has inflammatory infiltrates | 4 |
| **Quantity of perivascular infiltrates** | 0% of bronchioles with peribronchiolar infiltrates | 0 |
|  | 0-25% of bronchioles with peribronchiolar infiltrates | 1 |
|  | 25-50% of bronchioles with peribronchiolar infiltrates | 2 |
|  | 50-75% of bronchioles with peribronchiolar infiltrates | 3 |
|  | >75% of bronchioles with peribronchiolar infiltrates | 4 |
| **Severity of perivascular infiltrates** | No cells | 0 |
|  | interrupted cuff | 1 |
|  | Complete cuff, <5 cell layers thick | 2 |
|  | Complete cuff, >5 cell layers thick | 3 |
| **Quantity of interstitial infiltrates** | 0% interstitial infiltrates | 0 |
|  | 0-10% interstitial infiltrates | 1 |
|  | 10-33% interstitial infiltrates | 2 |
|  | 33-66% interstitial infiltrates | 3 |
|  | >66%interstitial infiltrates | 4 |
| **Quantity of alveolar infiltrates** | 0% of alveolar spaces with inflammatory infiltrates | 0 |
|  | 0-10% of alveolar spaces with inflammatory infiltrates | 1 |
|  | 10-33% of alveolar spaces with inflammatory infiltrates | 2 |
|  | 33-66% of alveolar spaces with inflammatory infiltrates | 3 |
|  | >66% of alveolar spaces with inflammatory infiltrates | 4 |
