# Supplementary material for: Immunogenicity and inflammatory properties of respiratory syncytial virus attachment G protein in cotton rats
Source: PLoS One. 2021 Feb 18;16(2):e0246770. doi: 10.1371/journal.pone.0246770 (PMC7891763; doi:10.1371/journal.pone.0246770)
Supplement: S3 Table — Five days after inoculation of cotton rats with 1010 DRP of AAV serotypes 1, 5, and 6 expressing GFP, tracheal cells were isolated and tested for GFP expression. GFP expression was evaluated against the autofluorescence background of tracheal cells from animals inoculated with PBS. Groups of three animals were used. (DOCX) [file pone.0246770.s007.docx]

**S3 Table. GFP expression in cotton rat tracheal cells after inoculation of different AAV serotypes.** Five days after inoculation of cotton rats with 10^10^ DRP of AAV serotypes 1, 5, and 6 expressing GFP, tracheal cells were isolated and tested for GFP expression. GFP expression was evaluated against the autofluorescence background of tracheal cells from animals inoculated with PBS. Groups of three animals were used.

| **AAV** | **Percent positve** |
| --- | --- |
| PBS | 12.4 ± 3 |
| AAV-1 | 15.3 ± 2 |
| AAV-5 | 20.9 ± 4 |
| AAV6 | 11.2 ± 4 |
