# Supplementary material for: Immunogenicity and inflammatory properties of respiratory syncytial virus attachment G protein in cotton rats
Source: PLoS One. 2021 Feb 18;16(2):e0246770. doi: 10.1371/journal.pone.0246770 (PMC7891763; doi:10.1371/journal.pone.0246770)
Supplement: Attachment — Submitted filename: PONE-D-20-27444_review.pdf [file pone.0246770.s008.pdf]

Immunogenicity and inflammatory properties of respiratory syncytial virus attachment G protein in cotton rats

Margaret E. Martinez, Cristina Capella Gonalez, Devra Huey, Mark E. Peeples, Douglas McCarty, Stefan Niewiesk

Summary:

This study explored the possibility that the RSV G protein alone induces pulmonary inflammatory responses. This study is motivated by the observation that antibodies against RSV G, or the deletion of the G gene from RSV, reduces inflammation during infection. The authors tested both purified RSV G protein and RSV G expressed by AAV in a cotton rat model and found no increase in pulmonary infection. Challenge with RSV showed decreased viral load in the lungs of animals that had previously received AAV-G or AAV-mG. There was significant correlation between high anti-G antibody levels as well as CD8+ Tcells and reduction of RSV load. Finally, mutation of the RSV G CX3C motif to CX3S eliminated its protective effects, suggesting that the structural integrity of RSV G is important for its protective effects.

Major comments:

1. The finding that RSV G alone does not induce inflammation does not necessarily mean that their data does “not support a role of the RSV G protein in inflammation” (line 462). The authors conclude from their data that anti-G antibodies block inflammation merely by reducing virus infection (lines 661-663), however it is still entirely possible that RSV G has a role in inflammation *in the context of RSV infection*. It is recommended to include this possibility in the discussion (lines 661-663) and also change line 462 to “…suggests that RSV G protein alone does not induce inflammation”

2. Throughout the manuscript, the authors describe the elicited anti-G antibodies as “non-neutralizing” because they do not inhibit RSV infection in HEp-2 cells. While this is a standard cell line for RSV growth, it is not necessarily an ideal model for RSV neutralization, especially for neutralizing anti-G antibodies. See for example https://pubmed.ncbi.nlm.nih.gov/26658574/ which shows that anti-G antibodies previously described as “non-neutralizing” are actually neutralizing in a relevant infection model (HAEs). It is recommended that the authors rewrite lines 489-490 “Only non-neutralizing RSV-G-specific antibodies were generated” to describe the caveat that the antibodies do not neutralize in this cell line, but it is possible that they neutralize in other cell types. To note, their own data supports that these anti-G antibodies are neutralizing (*in vivo*). We also recommend toning down the “non-neutralizing” statements throughout the manuscript (line 489, 532, 667, 678, 709, etc.)

Minor comments:

1. Studies with CX3CL1 use only the CX3CL1 chemokine domain, not the full ectodomain of CX3CL1, which could have different properties. This should at least be stated in the manuscript as a caveat to their studies.

2. Revise or eliminate lines 655-657 “This is in contrast to previous studies that suggested that the G protein is a chemoattractant causing increased pulmonary inflammatory infiltrates…” Similar to major comment #1 above, these studies do not necessarily contrast with previous studies. It is still possible that G protein is a chemoattractant causing increased pulmonary inflammatory infiltrates *in the context of RSV infection*.
