# Supplementary material for: Immunogenicity and inflammatory properties of respiratory syncytial virus attachment G protein in cotton rats
Source: PLoS One. 2021 Feb 18;16(2):e0246770. doi: 10.1371/journal.pone.0246770 (PMC7891763; doi:10.1371/journal.pone.0246770)
Supplement: Attachment — Submitted filename: Review of PONE-D-20-27444.pdf [file pone.0246770.s009.pdf]

Review of PONE-D-20-27444

This manuscript explores the role of the RSV G protein in lung inflammation, induction of total G protein antibodies, and protection from RSV challenge. The authors appropriately use cotton rats as an animal model and they express the G protein using an AAV vector. The manuscript shows data that challenges the view held by some in the field that it is the G protein that is responsible for lung inflammation after RSV infection. They found no evidence for induction of inflammation upon G expression in the animal lungs upon AAV-G intranasal inoculation. This conclusion is perhaps less convincing since their positive control, CX3CL1, also did not induce inflammation. They did however have a positive control, dust mite antigen.

To characterize antibody responses to the G protein, they used wild type G and mutant G proteins that expressed only the membrane bound form, the soluble form, or one defective in the receptor binding domain. They characterized total anti-G IgG, neutralizing antibodies, and levels of RSV in lungs and nasal tissue upon RSV challenge of the animals.

Major points

1. Conclusions based on absence of neutralizing antibody titers are puzzling. The authors used HEp2 cells in their neutralization assays. Cells that actually have adequate levels of CX3CR1, the G protein receptor, can be used to determine if anti-G antibodies can block virus entry (neutralize). Can the authors prove that HEp2 cells have adequate levels of the G protein receptor? Would the same results be found if primary human epithelial cells were used?

2. Comparisons of differential effects of wild type and mutant G proteins would be more convincing if the authors validated their constructions by showing proteins on gels, quantifying the relative levels of expression of the wild type and mutant proteins expressed from the AAV vector, and proving that the proteins were secreted or membrane associated or defective in CX3CR1 binding as predicted.

Minor Points

3. The authors should discuss why their positive control for inflammation, CX3CL1, did not cause inflammation.
4. Why did the methods include purifying G protein? This reagent was not mentioned in the results.
5. The numbers of animals/group used in some experiments were inadequate (3) or marginal (5). Cotton rats are not as inbred as mice thus larger groups are required for good statistics.
6. Lines 365-372 and Figure 2: discussion of the mutants should be in the same order as presented in the text.
7. Figure 3: how did the authors identify macrophages vs pneumocytes?
8. Line 455: sentence incomplete

9. In all figure legends, it is not clear if different points on graphs are the mean of different animals or the mean of multiple experiments on one animal. How many times were the determinations done?
10. Line 530: Do you mean figure 6, panels A and B?
11. Lines 701-703: this statement is contradictory. What is meant here?
